# 1.5 Decades Later: Bearing fruits from the ACR/EULAR exchange Program

**DOI:** 10.1186/1546-0096-9-14

**Published:** 2011-07-04

**Authors:** Randy Q Cron, Hendrik Schulze-Koops

**Affiliations:** 1 Department of Pediatrics, Division of Rheumatology, University of Alabama at Birmingham, USA; 2 Division of Rheumatology, Medizinische Poliklinik, University of Munich, Germany

## 

From its inception in 1997, the ACR/EULAR exchange program (AEEP) has brought young rheumatologists together from Europe and the USA. Over 40 junior physician-scientists and their respective senior rheumatologist mentors have travelled across the Atlantic Ocean to meet with and discuss science, medicine, and clinical practice patterns with their counterparts on the opposite side of the Pond. The format for the program of attending either the ACR Annual Scientific Meeting (the European colleagues) or the EULAR meeting (the American colleagues) followed by visiting several outstanding academic institutions in the week subsequent to the meeting has remained virtually unchanged since the beginnings of the AEEP [1]. However, with the current economic down-turnings, continued financial support for this program has been threatened. The survival of the AEEP should be considered in the context of its intended goals, namely creating a conducive environment for talented young rheumatology physician-scientists from Europe and America to learn from each other and form productive scientific collaborations.

One measure of success uniformly utilized in academia worldwide is quantity and quality of peer-reviewed scientific publications. To date, over 40 such publications have resulted from intra-continental collaborations between AEEP participants [e.g. [2]], and, perhaps more importantly, 40+ manuscripts have derived from inter-continental AEEP collaborative efforts [e.g. [3]]. The scientific topics of these collaborations have varied widely ranging from interferon-α and lupus [4] to mouse models of arthritis [5] to cartilage gene therapy [6] to genome wide association studies [7] to women in academic rheumatology [8], to textbook chapters [9], etc. The quality, quantity, and breadth of scope of these 80+ publications alone attests to the remarkable success of the AEEP in bringing promising junior physician-scientists from America together with their counterparts in Europe and vice versa.

Notable spin-offs from the AEEP that have also been quite successful have been the yearly AEEP Study Group sessions held at the ACR Annual Scientific Meetings and the corresponding "Forum for Young Rheumatologists" at the EULAR meetings. Because the AEEP group as a whole has a broad spectrum of research interests and scientific approaches this has allowed for a rather varied group of rheumatology related topics in these special sessions each year. The symposia have allowed for talks from the AEEP young investigators alongside lectures from invited experts in the field of study for the particular topic of that year's AEEP Study Group and Forum for Young Rheumatologists' meeting. Previous topics and speakers in the recent past have included the following: "Synoviocyte and NK T Cell Signaling" with Drs. Gary Firestein (University of California, San Diego) and Mitchell Kronenberg (La Jolla Institute for Allergy & Immunology), "Regulatory T Cells and Autoimmunity" with Drs. Ethan Shevach (National Institutes of Health) and Berent Prakken (University Medical Center, Utrecht), "Aging and Autoimmunity" with Drs. Bruce Richardson (University of Michigan) and Cornelia Weyand (Emory University), "Endothelium and Autoimmunity" with Drs. Ulrich von Andrian (Harvard University) and Umar Mahmood (Harvard University), and most recently, "Interleukin-1 and Related Molecules" with Drs. Hasan Yacizi (University of Istanbul) and Cem Gabay (University Hospitals of Geneva), as well as "B Cell Targets in Rheumatology" with Dr. Peter Lipsky (formerly at the NIH). Whether or not the AEEP continues on as originally intended (as an exchange program), it is likely the study group sessions will continue for the excellence of the content presented by the junior and more senior lecturers.

If the ACR and EULAR decide to re-establish the exchange program portion of the AEEP, the success of the venture will continue to rely heavily on the quality and enthusiasm of the selected junior faculty physician-scientists, but will also be dependent on outstanding host faculty and institutions. Some 14 years after its implementation there have been no less than 16 (Drs. Aringer, Colbert, Cope, Cron, Diakh, Elewaut, Ferguson, Huizinga, Muller-Ladner, Oates, Pap, Schulze-Koops, Schanberg, Singer, Voll, and Von Scheven) of its approximately 40 AEEP "junior" faculty participants who have proceeded to rheumatology directorships at prestigious institutions in the USA and Europe. From the American side, a disproportionate number of pediatric rheumatologists (6 out of 15 participants, or 40%) have benefited from this program, and all have gone to directorships. This degree of academic success may in part reflect the selection process for participation in the exchange program but also likely demonstrates the benefits accrued from participation. The exchange program likely would have benefited from involvement of pediatric rheumatologists from Europe, as well as pediatric rheumatologists serving as senior faculty participants. Moreover, most all the former junior faculty AEEP participants, both adult and pediatric rheumatologists, have gone on to highly successful academic careers and have taken on leadership positions in rheumatology spanning the Atlantic Ocean as the founders of the AEEP could only have hoped for when the program was first contemplated. This seems to have been a rather strong return on investment for the ACR and the EULAR.

If, on the other hand, the American/European exchange portion of the AEEP comes to an end, it was the privilege of all the former participants to have been involved in this ambitious endeavor. For most all participants, junior and senior scientists alike, participation in the AEEP was a stepping stone to further and greater academic success, a camaraderie of sorts, a source of many and varied rich scientific collaborations, and, if nothing else, a conduit to long lasting friendships bridging the Atlantic Ocean.

## References

[B1] Elewaut D Aringer M Muller-Ladner U Getting scientists together: the ACR/EULAR exchange programme Ann Rheum Dis 2003 62 8 789 90 10.1136/ard.62.8.789 12860744 PMC1754634

[B2] Eskdale J Gallagher G Verweij CL Keijsers V Westendorp RG Huizinga TW Interleukin 10 secretion in relation to human IL-10 locus haplotypes Proc Natl Acad Sci USA 1998 95 16 9465 70 10.1073/pnas.95.16.9465 9689103 PMC21361

[B3] Huizinga TW Amos CI van der Helm-van Mil AH Chen W van Gaalen FA Jawaheer D Schreuder GM Wener M Breedveld FC Ahmad N Lum RF de Vries RR Gregersen PK Toes RE Criswell LA Refining the complex rheumatoid arthritis phenotype based on specificity of the HLA-DRB1 shared epitope for antibodies to citrullinated proteins Arthritis Rheum 2005 52 11 3433 8 10.1002/art.21385 16255021

[B4] Aringer M Crow MK A bridge between interferon-alpha and tumor necrosis factor in lupus J Rheumatol 2008 35 8 1473 6 18671317

[B5] Dewint P Gossye V De Bosscher K Vanden Berghe W Van Beneden K Deforce D Van Calenbergh S Müller-Ladner U Vander Cruyssen B Verbruggen G Haegeman G Elewaut D A plant-derived ligand favoring monomeric glucocorticoid receptor conformation with impaired transactivation potential attenuates collagen-induced arthritis J Immunol 2008 180 4 2608 15 18250472 10.4049/jimmunol.180.4.2608

[B6] van der Laan WH Quax PH Seemayer CA Huisman LG Pieterman EJ Grimbergen JM Verheijen JH Breedveld FC Gay RE Gay S Huizinga TW Pap T Cartilage degradation and invasion by rheumatoid synovial fibroblasts is inhibited by gene transfer of TIMP-1 and TIMP-3 Gene Ther 2003 10 3 234 42 10.1038/sj.gt.3301871 12571631

[B7] Raychaudhuri S Remmers EF Lee AT Hackett R Guiducci C Burtt NP Gianniny L Korman BD Padyukov L Kurreeman FA Chang M Catanese JJ Ding B Wong S van der Helm-van Mil AH Neale BM Coblyn J Cui J Tak PP Wolbink GJ Crusius JB van der Horst-Bruinsma IE Criswell LA Amos CI Seldin MF Kastner DL Ardlie KG Alfredsson L Costenbader KH Altshuler D Huizinga TW Shadick NA Weinblatt ME de Vries N Worthington J Seielstad M Toes RE Karlson EW Begovich AB Klareskog L Gregersen PK Daly MJ Plenge RM Common variants at CD40 and other loci confer risk of rheumatoid arthritis Nat Genet 2008 40 10 1216 23 10.1038/ng.233 18794853 PMC2757650

[B8] Lundberg IE Ozen S Gunes-Ayata A Kaplan MJ Women in academic rheumatology Arthritis Rheum 2005 52 3 697 706 10.1002/art.20881 15751096

[B9] Schulze-Koops H Skapenko A Cope AP Immunology and the Rheumatic Diseases in the EULAR Compendium of Rheumatic Diseases 2009 Chapter 4 Zurich

